# High use of over-the-counter analgesic; possible warnings of reduced quality of life in adolescents - a qualitative study

**DOI:** 10.1186/s12912-016-0135-9

**Published:** 2016-03-03

**Authors:** Siv Skarstein, Per Lagerløv, Lisbeth Gravdal Kvarme, Sølvi Helseth

**Affiliations:** Department of Nursing, Oslo, and Akershus University College of Applied Sciences, 4, St. Olavsplass, Oslo, NO-0130 Norway; Department of General Practice/Family Medicine, Institute of Health and Society, The Medical Faculty, University of Oslo, Oslo, Norway

## Abstract

**Background:**

Use of over-the-counter analgesics among adolescents has increased markedly. High consumption of over-the-counter analgesics among adolescents is associated with frequent pain, lower self-esteem, reduced sleep, lower educational ambition, binge drinking, higher caffeine consumption, and part-time employment. Knowledge about life experiences of adolescents who frequently use over-the-counter analgesics may be useful to prevent health problems. The purpose of the study was to increase knowledge about adolescents who suffer from frequent pain and have a high consumption of over-the-counter analgesics.

**Methods:**

A qualitative study, employing one-on-one, in-depth interviews using a thematic interview guide. Data were collected in Norway in 2013–2014. Three boys and sixteen girls; aged 14–16 years, who continuously consumed over-the-counter analgesics were recruited from ten high schools in urban and suburban districts. Candidate participants were excluded if they were medically diagnosed with an acute or chronic illness, requiring extended use of over-the-counter analgesics within the last year. The interviews were taped, transcribed and analysed as text according to Kvale’s three contexts of interpretation: self-understanding, common sense and theory.

**Results:**

All participants disclosed unresolved physical and psychosocial distress characterized as pain. Frequent pain from various body parts made everyday life challenging. Methods of pain self-appraisal and over-the-counter analgesics use often mimicked maternal patterns. Participants reported being raised under unpredictable circumstances that contributed to long lasting family conflicts and peer-group problems. Participants wanted to feel appreciated and to be socially and academically successful. However, pain reduced their ability to manage everyday life, hampered experienced possibilities for success, and made social settings difficult.

**Conclusions:**

Childhood experiences influence how adolescents experience pain and use over-the-counter analgesics. Coping with difficult situations or attempting to mask symptoms with over-the-counter analgesics can perpetuate and amplify underlying problems. High consumption of over-the-counter analgesics and frequent pain may be warning signs of adolescents with possible health threatening conditions and reduced quality of life. These adolescent might be in need of support from school nurses and General Practitioners. This study identifies new perspectives that may lead to novel approaches to identify, guide, and support adolescents with frequent pain and high consumption of over-the-counter analgesics.

## Background

Adolescents, aged 12–17, frequently use over-the-counter analgesics (OTCAs) to alleviate common ailments and Norwegian studies shows that during the last two decades, this use has increased markedly [1–3]. The most selling OTCAs in Norway are paracetamol and ibuprofen [4]. Similar tendencies are found in Sweden, Denmark and in the United States [1, 5, 6]. OTCA medicine abuse, broadly defined as the systematic overuse of non-prescription medicine [7], is a serious global health challenge [8]. High consumption of OTCA among adolescents is associated with frequent pain, lower self-esteem, reduced sleep, lower educational ambition, binge drinking, higher caffeine consumption, and part-time employment [9]. These adolescents’ ability to handle stress appears to be discordant with the kind of situations to which they are exposed, and the wear and tear associated with allostatic mechanisms counteracting stress may heighten their pain experience [9]. In general an increasing number of adolescents state they experience frequent pain [10]. Anxiety and depression show a strong relation to chronic pain and may contribute to the development and, more surely, to the preservation of pain [11]. OTCAs may also be a preferred way for some adolescents to deal with perceived health problems [12], and some adolescents use OTCAs to treat stress [13].

Childhood is important for attachment, development of identity, and learned strategies for coping with stress and pain [14–16]. Research suggests that familial methods of managing pain are evident in adolescents’ OTCA use; intergenerational transmission of information about pain and pain management has been traced to maternal domiciliary practices [17]. Early identification and targeted interventions for adolescents challenged by chronic pain and overuse of OTCAs may result in decreased pain-related disability and improved quality of life (QOL) [10, 18]. WHO defines QOL as: ‘*the individual’s perception of his or her position in life, within the cultural context and value system he or she lives in, and in relation to his or her goals, expectations, parameters and social relations. It is a broad ranging concept affected in a complex way by the person’s physical health, psychological state, level of independence, social relationships and their relationship to salient features of their environment* [19]*.* Most researchers agree on some characteristics of the concept QOL [20] and one point of consensus is that QOL is a multidimensional concept, which includes the physical, psychological, and social dimension of life. Secondly, QOL is a subjective phenomenon, insofar as all individuals have their own perspectives on their well-being and their lives. Thirdly, QOL is a normative concept based on the individual’s expectations, values, goals and understanding of the meaning of life [20, 21]. Quality of life when young is the basis for quality of life in adulthood [22]. Thus, in this paper, the adolescents’ life circumstances and family problems will be in focus.

Recent studies have focused on the increasing prevalence of pain and the concomitant rise in the use of OTCAs among adolescents. However, little research has sought to expand understanding from the perspective of adolescents with frequent pain who also consume potentially harmful amounts of OTCAs.

### Purpose of the study

The purpose of this study was to gain a deeper understanding and increase our knowledge about adolescents who suffer from frequent pain and have a high consumption of OTCAs. Such knowledge may aid school nurses and general practitioners (GPs) in the identification of adolescents at risk for chronic pain, continuous and high use of OTCAs, and in finding effective interventions.

## Methods

This study has a qualitative design, with qualitative in-depth interviews of adolescents, to provide new knowledge, insights, and representations of common issues as a practical guide to future research and intervention.

We used a convenience sample based on participant self-selection. Principals in 20 randomly chosen schools representing; urban, sub-urban and rural districts in South of Norway, were by phone invited to participate. Principals of ten schools gave written consent to inform and recruit adolescents at their schools. One of the researchers visited these ten schools and gave a 15 min presentation of the project to all classes in 9th and 10th grades, a total of 52 classes with an average on 25 students in each. The adolescents were given written information about the project including a study information package with a combined bookmark/ruler that contained brief information about the study and the researchers’ contact information. This information package also included information for the parents. The adolescents did not receive any compensation for participation.

Eligible participants included students between 14–16 years of age using OTCAs either daily, or on a one- to several times a week schedule, for at least four continuous weeks within the last year. Participants were required to be able to read and understand the Norwegian language. Exclusion criteria were being medically diagnosed with an illness or injury requiring extended use of OTCAs within the last year.

The research group developed and employed a thematic interview guide addressing the adolescents’ life history and family life, OTCA usage, pain, stress, coping, peer relations, eating habits, sleep, activities, and important life events. However, participants were given the opportunity to steer the conversation so that their stories and experiences appeared spontaneously [23, 24]. Interviews were carried out from September 2013 until March 2014. Participants were recruited and interviewed until we were confident that no new themes were emerging [25]. One researcher (the psychiatric nurse) conducted the one-on-one interviews, lasting approximately one hour, at an office at the adolescents’ schools or at an office at the researcher’s work place.

The interviews were audio recorded and transcribed verbatim. The audio files and the transcribed material were uploaded within the Nvivo 10 computer program [26] for organization, review, and analysis.

A hermeneutic approach was taken in the analysis, involving an interpretive circular process of moving back and forth between the raw data and the interpretations [27]. In the analysis process, the researchers listened to audio recordings, and read and re-read the transcripts. The analysis was done in three different contexts: self-understanding, common sense and theory [28]. In the first context, the transcribed interviews were read and summarised in a condensed form, following broad categories that emerged from the data, such as: family life and childhood, friendship, school life, about myself, pain, health, use of OTCAs, important life events, support from professionals, quality of life, health, self-harming and suicidal tendencies. All interviews were then reread and coded, after which categories were assigned using descriptive terminology; for example: ‘family and childhood’. The next step was to interpret the meaning of these findings. During the interpretation patterns emerged that form the basis for the presentation of our findings*:* Table 1 outlines the analysis process.

**Table 1:** Analysis of the adolescents’ expressions obtained from thematic, semi-structured interviews^a^

Self-understanding	Common sense	Theoretical understanding
How does the participant reflect and understand his or her own expressions?	How do people, in general, think critically about the statement below?	A scientifically tested assumption of an expression as a phenomenon or in connection to nature.
‘The stress makes me really tired, and then it becomes too much for me; then it is okay with painkillers. It is a nice solution to stress’.	‘Something is experienced as stressful in the adolescent’s life. Stresses makes the adolescent feeling tired. Painkillers are used to cope with the feeling of stress’.	‘Successful adaptation to stress includes the ways in which individuals manage their emotions, think constructively, regulate and direct their behaviour, control their autonomic arousal, and act on the social and non-social environments, to alter or decrease sources of stress’ [29].

^a^The adolescents’ statements were analysed in terms of their self-understanding, common sense, and within the context of applicable theories, as outlined by Kvale and Brinkman [28]

Group consensus usually emerged for most of the interpretations. When any doubt or disagreement arose, the researchers went back to the data, reassessed the meaning of the participant’s expressions, and brought this into new discussions. Through this repetitive process, a broader understanding emerged. In the third context, the findings were interpreted and discussed in the light of theories of adolescent development research and QOL.

### Trustworthiness

Our research group, consisting of a general practitioner, two public health nurses, and a psychiatric nurse, considered a qualitative design with a hermeneutic approach to be suitable in order to achieve trustworthiness of our findings [29]. Activities that ensured methodological coherence, sampling sufficiency, a relationship between sampling, data collection, analysis, and theoretical understanding, were discussed at regular meetings in the research group [30].

To give an accurate picture of the adolescents’ expressions, we present representative quotes from the interviews in the results. These quotes were viewed as meaningful units and were extracted directly from the transcripts and translated into English, and then back into Norwegian by an English-Norwegian translator. Importantly, we chose a girl, who was 17 years of age and familiar with the style of language the adolescents used, as translator.

### Consent and ethical considerations

The Regional Committees for Medical and Health Research Ethics (REC) in Norway, approved the study in autumn 2012, study number: 2012/1460a. Data and material from the study are kept in the department of Nursing, Oslo and Akershus University Collage of Applied Sciences, Oslo, Norway. The ethical principles highlighted in the Declaration of Helsinki [31] were followed. Participation was voluntary and required written informed consent. For participants younger than 16 years of age, written informed consent from their parents was required.

In each school, the school nurse was informed about the study and was prepared to support adolescents at any health risk. By signing a consent form, the adolescents also agreed to consult the school nurse if the interviewer deemed it necessary. The participants were informed that they were free to withdraw from the project at any time.

## Results

The sample consisted of 19 adolescents, ranging from 14 to 16 years old. Participants were recruited from the ninth and tenth grades in five of the ten participating high schools in Norway. Selected demographics and psychosocial problems of the 19 interviewed adolescents are shown in Table 2.

**Table 2:** Selected demographics and psychosocial problems of the 19 interviewed adolescents^a^

Demographics		Number
Gender	Male	3
	Female	16
School location	Urban	8
	Rural	11
Living with	Both parents	8
	One parent	6
	Part-time with each parent	4
	Other caregivers	1
Psychosocial problems		
Required support from Child Protective Services	Yes	6
	No	13
Experienced eating problems	Yes	6
	No	13
Experienced persistent bullying	Yes	9
	No	10

^a^The subjects’ ages ranged from 14 to and including16 years

In addition to frequent pain, having needed support from Child Protective Services, having experienced persistent bulling and having had eating problems was surprisingly common.

The adolescents reported pain in several parts of the body; almost all reported frequent headaches. The pain reduced their ability to manage everyday life, hampered experienced possibilities for success, and made social settings difficult. The participants had lived with their pain for a long time, some said they have had pain from childhood, others said they had recognized pain problems from beginning of adolescence. None reported that any treatments had brought lasting relief. The adolescents often described serious problems within their family and peer groups. Several exerted much energy to conceal personal and family problems. To appear successful, the adolescents valued excelling on tests and exams, as well as achieving success in sports, art, and culture. They felt that such success was essential in order to feel good. Furthermore, looking good and being popular among their peers were considered significant. They all struggled to fit in with their peers and to feel valued. The main theme emerging from the adolescents’ stories could be summed up in the following statement: *High consume of OTCAs and pain in everyday life: warnings of reduced QOL in adolescents.* The following three sub-themes elaborate on the overall theme:

- Pain and use of OTCAs
- Being raised under unpredictable conditions
- Wanting to feel appreciated and appear successful

These three sub-themes are not mutually exclusive; still, we chose this tripartite division to organize and structure the diverse and rich information the adolescents shared with us during their interviews. The numbers in parentheses following the quotations refer to the subject’s transcribed interview.

### Pain and use of OTCAs

The adolescents said they experienced continuous pain over years, one said she had had stomach pains from she was about 4 years old. They all stated that their pain problems had escalated. Generally, the adolescents said their pain was located in different parts of the body. It might have started in one part of the body, but had spread to other parts. Nearly all reported suffering from headaches, and that these were especially troublesome. The adolescents said they struggled to understand the reason for their pain and to rate their pain, some said further that they were reluctant to bother others with complaints. For example, one girl stated that she was so used to having stomach pains that she did not disclose this fact prior to her departure on a trip. After being brought to hospital following a fainting episode during her trip, the doctors found she needed immediately surgery, due to a life threatening and very painful condition.

The majority of participants reported that they had visited different kinds of doctors and health professionals because of pain or fatigue. They often received a tentative diagnosis, supporting their feeling of something being medically wrong. This seemed problematic as it left the individual in a liminal place relative to a solution. One girl explained:

‘Actually, I’m often sick because my immune system is weak. When I went to the doctor, I found out that I had allergies, so that could be one of the reasons. I often have headaches, as well as being tired a lot. I often think that it’s allergy, but you never know… My shoulder got inflamed in seventh grade in gym class… We went to the doctor, and he almost laughed because it hurt so much, but then they found out that I had an inflammation. So we went to a physical therapist and an osteopath’. (P.14)

Several mentioned stress as a cause of their pain. Like this girl, some said they used OTCAs to combat stress and fatigue:

‘Stress makes me really tired, and then it gets to be too much for me, and so it’s nice to take a painkiller. It’s a nice solution to stress’. (P.6)

Some said they felt better by taking OTCAs before a potentially challenging situation, such as a social event, exams, athletic training, and competitions. Some said they used OTCAs both to calm down and to keep focused. OTCAs were described as a tool for both preventing and managing pain, stress, and fatigue. The following girl used OTCAs to feel better when dating a boyfriend:

‘I actually had a boyfriend last year. Every time I met him, I would take an ibuprofen before I left so I wouldn’t feel so tired when I was with him. Then I was like more happy and alive… I felt that it helped a lot, but it was probably psychological’. (P.6)

The adolescents disclosed a sense of shame about their OTCA use. Most of them stated that lately their OTCA use had diminished markedly. Others said they hoped to reduce OTCA use in the future. Eventually, it emerged that they still used OTCAs to a rather great extent, even if they reported experiencing little or no effect, as shown by this girl’s statement:

‘Earlier I took painkillers three times a day … or two. It depended on how the school day was. But now I have reduced my consumption a lot. Yeah, so the question is what I have in class at school. If I had a double language session, that can be quite boring, so I take two pills and then I’ll also have a little snack. I took a dose when I came here. I did not take anything yesterday… I do not think I took any yesterday. Yes, before my French class I took some pain medication. But otherwise it has been reduced. Perhaps about every second day I’ll take one or two doses. (P.4-girl)’.

Without access to OTCAs, several participants said they felt unsure about whether they could manage daily life. The adolescents sometimes both justified and trivialized their OTCA use, mentioning uncertainty about the source of pain:

‘If it wasn’t for the fact that the pain medication worked, I would have thought my pain was psychological. It is like when I am in pain, I make myself believe that the pain is much worse than it actually is. I have a very low pain threshold. Everything hurts all the time. I think that I make myself believe it hurts, even more than it actually does. I think that by taking the medication I am telling myself that the pain is so bad that I need medication, and if I need medication, then the pain must be bad. I end up thinking I’m in more pain than I actually am. If there were no pain medication, I think that I would feel a lot less pain’. (P.2-girl)

Almost all said they talked mainly to their mother about pain and several said that their mother and maternal ancestors had similar problems. Several adolescents said their mothers supported them in the use of OTCAs and sometimes mothers shared their own prescribed pain medication. Mothers also initiated help-seeking for pain from public and private health professionals on behalf of their adolescents.

### Being raised under unpredictable conditions

The majority of the adolescents had experienced a disrupted family life with sadness and grief and this had influenced their childhood. Worries about others’ well-being were common. Some had incompetent caregivers and a childhood characterized by feelings of unpredictable danger, worries, and uncertainty. Others grew up in a climate in which the family expressed feelings of sadness. The following quotes from two girls illustrate this range of feelings. The first girl explained how she had to protect herself and a younger sister from a drugged and mentally ill mother after they were placed in another home by the Child Protective Service:

‘… my mother broke into our house and demanded to get us back. This happened on multiple occasions. One of the times she came, I took my sister and hid under the table. Then I covered her eyes, and she had to cover her own mouth so she wouldn’t start screaming. I had to sort of shade my own eyes and ears, because I couldn’t cover them myself since I had to cover hers…’ (P.15).

Another girl explained the family climate like this:

‘I’ve never felt unsafe, but I have often been sad, because there have been many conflicts. Mom has been sad and mom has been mad. Dad talked badly about mom, and mom talked badly about dad. I have often felt sad’. (P.5)

Another theme mentioned was frequent changes of residence, either temporarily or permanently. Some moved weekly between their parents and described this as stressful. Some said this repeatedly disrupted ties to peers. Moving was one reason given by some for feeling that they did not fit in. One of the boys reflected on how this resulted in him being bullied:

‘I think we moved eight or nine times, or something like that. I was bullied in elementary school, most likely because of all the moving, so I was probably affected a lot by it’. (P.7)

Several of the adolescents spoke about challenges caused by broken homes and forming new family relationships involving a diversity of biological and non-biological parents and siblings. Illnesses and deaths of close relatives causing long lasting worries, insecurity, sorrows and grief were often mentioned by some of the adolescents. Several, like the following boy, described living with a mentally unstable parent who abused alcohol and other substances:

‘He wasn’t normal or like normal people. He couldn’t control himself and was out of control, and sometimes he got really pissed or angry’. (P.19)

This same boy had learned strategies in childhood to protect himself:

‘When I’d come home in the evening, my dad was often drinking. If I noticed that, I usually biked to a friend’s house. Then I’d tell my dad that I made plans to sleep over at a friend’s house, and then I’d go over to someone else’s and sleep there. I’d feel good for having avoided a big problem, because I never knew what would happen’. (P.19)

A memorable aspect of the adolescents’ childhood was that their parents were preoccupied with their own careers or had a heavy work-load caring for disabled relatives. According to the adolescents, several parents had health problems, especially mothers. The adolescents’ stories revealed that many of them received little or no help from their extended family. One of the girls had recurrent nightmares that might be connected to a feeling of needing help, but not getting it:

‘I dreamt that I got killed, and my family watched without helping. I try to forget it, but it always comes back, so I wouldn’t dare sleep’. (P.10)

Despite their difficulties at home, the adolescents were very loyal to their parents, and it was important for them to be able to make their parents proud. Outside the home, they tried to conceal problems within the family and felt threatened when their family problems were revealed. For some of the adolescents, it was difficult to establish close friendships for fear of exposing their problems at home. If a conflict arose between one’s own desire for friendship and protecting family secrets, family loyalty took precedence. Some related stories of their loyalty to each parent being tested when their parents were in conflict.

### Wanting to feel appreciated and to appear successful

Throughout the interviews, it became clear that the adolescents pressured themselves intensely to perform well, especially on visible tasks. This sentiment was clear in this girls’ statement:

‘I am happy with my grades, but I want to focus more and not be so passive. It’s easy for me to postpone things; actually I postpone most things. I want to learn to do things and not postpone them to the last minute. I get mostly 5’s in all my subjects; in some I get 6’s [a score of six is the highest score attainable]. I’m mostly between a 5 and a 6, so I’m not happy’. (P.18)

Most of the adolescents were preoccupied with attaining and maintaining a good appearance, such as expressing polite behaviour, having fit bodies, and dressing properly. Several, like this girl, expressed dissatisfaction with themselves and said they often tried to change their appearance through intensive dieting and exercise:

‘It was mostly that I didn’t like who I was, so I tried to improve my confidence by dieting… that was when I started using painkillers’. (P.6)

The adolescents said they strived for acceptance, and they tried to comply with others’ expectations, like this girl:

‘Now I’m focusing on doing well in school as well as trying to remember everything I need to do at home. I still want to be with friends, though. I’m trying, but it’s hard living up to everyone’s expectations’. (P.4)

The adolescents needed to maintain control, which was experienced as stressful, as expressed by one girl:

‘I’m not one who gives up easily. I don’t give up, and I’m really sort of stressed … I feel I always need to have control’. (P.10)

Many of our adolescents were extremely attentive to signals indicating social conflict within their peer group. To avoid conflicts some, like this girl, lay awake at night thinking about what to say and how to behave.

‘You never know what the other girls are saying behind your back, so you go around thinking about it’. (P.13)

Almost every adolescent in this study mentioned a fear of getting involved in social conflicts, both at home and at school. Tremendous energy was expended trying to avoid these difficulties. Several, like the girl quoted below, had been bullied and learned to avoid conflicts:

‘I’ve had conflicts with one of the girls, and it’s like social suicide if you become her enemy’. (P.11)

Declining to take part in conflicts, even when a friend is bullied, was experienced as being cowardly. Yet, this might be necessary. Some expressed an inner feeling of guilt and a pressure to speak up. At the same time, they didn’t want to attract attention. A girl explained how she suppressed her feelings:

‘I don’t really like conflicts, so I often say what other people want to hear. I hate it when I feel like people don’t like me’. (P.18)

In several of the adolescents’ stories, their feelings of loneliness and their desire to put on a successful facade eventually emerged. In the beginning of the interviews, the adolescents mostly related their ‘success’ stories. This included telling the interviewer about their many friends, as revealed by this girl:

‘I have many friends here as well as many from all over Norway, because I’ve moved around so much’. (P.15)

Later on in the interviews, many participants expressed a longing for a close friend, one they could trust, be open with, and reveal their real feelings to without putting on a facade of a ‘successful’ appearance. Mostly, the adolescents kept hoping that they would find a good friend, like this girl:

‘I don’t feel like I have a good friend, someone that I can tell everything to … I have a good feeling that I will find this friend in high school’. (P.3)

Some of the adolescents had thought about taking their own lives; a few engaged in self-harm such as cutting themselves. Many said that they had been rejected by parents and/or friends, and this left them in a very fragile state, as shown in the following excerpt from one of the girls’ interview:

‘I thought nobody liked me, and there was no reason to live, because I was going to die someday anyway. I did not want anyone to help me. I felt afraid and ashamed. I did not dare tell my parents. I had no need to tell anyone’. (P.11)

The findings presented illustrate how the need to feel appreciated and appear successful cause stresses that affect the use of OTCAs, as this girl expresses:

‘I have always felt quite different really… never felt that I’ve fitted right in. In lots of situations I’ve felt I must adapt myself properly… and it’s like the worst feeling in the world to me. Now I’m really just trying to be myself, and it works much better. It is so difficult to adapt to everything. You get really tired of it and very stressed. It’s so tiring beyond the teens. I think there are lots of people who feel that way, girls at least. So I do not think it was a coincidence that just I went on painkillers. Yeah, it sounds like I’m talking about drugs here, but somehow painkillers is something you feel is well known, and they’re available without prescription, so they’re so easy to get and a very easy solution at things’ (P.12).

In this and other instances, the availability and perceived safety profile of OTCAs reinforced ongoing analgesic usage. The lack of definitive medical solutions or alternative interventions for ongoing pain, unpredictable family circumstances, and social stressors motivated adolescents to take OTCAs both preemptively and during pain precipitating events.

## Discussion

The adolescents in the present study experienced complex intersecting problems including moderate-to-severe pain, fatigue, sleeping problems, lack of concentration, feelings of loneliness, stress, worry, sadness, and anxiety. They struggled hard for success in various areas and had high expectations for their achievements. Most reported serious family problems throughout their childhood. As an illustration of the load of psychosocial problems described, many also talked about serious eating problems, more than half had been bullied and almost one third had required support from the Child Protective Services. The adolescents described continuous feelings of not being good enough. In addition, they missed having a close friend. The adolescents used OTCAs to deal with the various problems, even though the problems often were outside the known medical indications for OTCAs. Most participants had tentative or incomplete medical insights into their pain leading to uncertainty and rationalization regarding their need for OTCAs. According to the adolescents, mothers were the prime care givers throughout their childhood and the mothers engaged naturally in caregiving to alleviate pain and improve their child’s health. Several had transitioned from mother-assisted OTCA use to become more autonomous.

### Pain is a complex concept

The adolescents stated that they suffered from severe pain and that understanding the source of their pain was difficult. Some were unsure whether their ‘pain’ was really pain. Some linked pain to stress. Others described how they may have manipulated themselves by focusing exhaustively on bodily sensations, thus giving themselves a reason to use OTCAs. In our analysis, the word ‘pain’ seemed to be a proxy for adolescents to describe their feelings when these became troublesome, regardless of the source. In the biomedical literature, pain is defined as ‘an unpleasant sensory and emotional experience associated with actual or potential tissue damage, or described in terms of such damage [32]. Pain can also be characterized in more personal terms as ‘whatever a person who feels pain says it is and may not be caused by external, physical trauma’ [33]. While these definitions are helpful, they understate the subjective experience of pain by emphasizing bodily pathology. The aetiology of chronic or persistent pain is often unknown, and a complete understanding of pain must take into account biological, psychological and social factors to achieve a bio-psycho-social understanding [34]. In expanding conceptualization of pain, Flor & Turk [35] suggest that pain affects the whole person and is regarded as more than a sensation or a symptom of a disease. This underscores that defining the concept of pain completely constitutes a challenge [36]. However, interpreting one’s difficulties as ‘experiencing pain’ gives adolescents access to something they can use to treat their troublesome life, namely OTCAs. OTCAs are suitable for treating various types of pain [37], but the medication can also cover signals of underlying health conditions. In the present study, adolescents acted to conceal their personal and family problems, and they seemed to have learned that pain is an accepted way to express their discomfort with life experiences.

### Childhood experiences, stress, and pain

The current study shows how participants’ individual needs for establishing predictability, stability, attention, and continuity were not properly met [38, 39]. Child development is shaped in the interplay between genetic and environmental influences [40]. Children form attachments to their caregiver, even if the caregiver is insensitive and unresponsive during social interactions [16]. Open communication of pain effect, inhibition of pain effect, and exaggeration of pain effect may reflect adaptations to different relationship contexts. This has important implications for an individual’s future life experiences, because experiencing attachment shapes how a child expresses pain to caregivers and later also to others [41]. In the present study, expressing pain may have been an acceptable way for these adolescents to express needs beyond physical discomfort, needs that resulted in attention and care. The types of pain experienced have been described as being inherited [42]. Expressing pain may be a well-known characteristic of family members; thus, when adolescents express pain, a sense of belonging to the family may result. Furthermore, pain can be a learned and acceptable way to express negative thoughts and emotions. Associations have been found between depression, pain, and functioning [43]. Over time, this pattern may influence appraisal and coping [44]. Maternal OTCA use is associated with child self-medication with OTCAs [45]. Mechanisms by which mothers transmit information to adolescents about pain and pain management primarily involve verbal communication and modelling [17]. Shared attitudes and pain-management strategies underscore the role of mothers as models and essential facilitators of their adolescents’ transition towards autonomy in pain management [46]. The complexity of attachment, ways of communicating, and ways of coping should be considered to be important factors in understanding and supporting adolescents who experience frequent pain and have a high consumption of OTCAs. Demands and threats placed on adolescents early in life can be hard for them to handle, especially when neither their family nor their social network have resources to intervene. Early life experiences are intimately embedded into the regulation of stress systems in ways that shape an organism’s future responses [40]. Adverse or unfavourable childhood experiences can make adolescents vulnerable to challenges and stresses later in life [47]. Increased demands on adolescents with unstable and unpredictable childhood experiences might over time lead to allostatic overload with increased pain sensitivity [48]. This seems to be one possible explanation for the persistent pain experiences expressed in the interviews. Adolescents in the present study said they felt intense social pressure resulting in stress and fatigue. Successful adaptation to stress includes the ways in which individuals manage their emotions, the ability to think constructively, the ability to regulate and direct their behaviour, the ability to control their autonomic arousal, the way they act in social and non-social environments, and the ability to alter or decrease sources of stress [49]. High consumption of OTCAs may be an adapted behaviour which vulnerable adolescents in general use to manage life challenges and stresses [50]. Others have found that teenagers appear to have an attitude towards OTCAs that ranges from casual to careless, and some use OTCAs for their sedative effects [51]. Our analysis supports these findings.

### Managing pain: influence of social acceptance

The adolescents described experiencing relationship problems with parents and friends. Almost all struggled intensely to fit in, and several were bullied. Unsatisfactory social relations are associated with feelings of loneliness [52, 53]. At the same time, the adolescents sought to present an ideal picture of themselves and to make a good impression. To accomplish this, it was important to establish a positive self-image. Good relations with peers and parents preserve a positive feeling of one’s own value, social position, and competence [54]. Problems with establishing and maintaining relationships during adolescence may be related to being raised in unstable families [55]. Reports of feelings of fear and anxiety connected to events considered as stressful were common; thus, the adolescents in this study made great efforts to avoid conflicts and to control unpredictable situations and challenges. The adolescents said that they avoided speaking up, even though they wanted to. Avoiding conflicts has been described as both an automatic, involuntary response and a controlled, voluntary response [56]. To the adolescents in our study, controlling both behaviour and emotions was important in order to hide their vulnerabilities. This may indicate low self-esteem and low self-efficacy, which are also associated with anxiety and depression [57, 58]. The relative contribution of behavioural and cognitive responses varies, depending on the stress context, the adolescents’ developmental level, and how they have learned to respond to stress [56]. Expressing emotions as pain may be a learned, controlled and accepted way our adolescents used to vent their feelings.

### Life experiences and QOL

Most of the adolescents expressed dissatisfaction with themselves, their appearance, and their achievements. They seemed to have learned to conceal their inner feelings in order to be accepted and included both in the family and among peers. According to adolescents in another Norwegian study, specifically feeling good, being satisfied with oneself, having an overall positive attitude, and a positive self-image were important for having a good QOL [53]. Adolescents’ self-image is associated with the ability to make friends [59], and good family relations are further important for QOL [53, 60]. Most of the adolescents in our study had experienced both family conflicts and problems in peer relations. Several had been bullied over time, which is associated with psychological health complaints such as depressive symptoms, psychological disturbance and with pain problems [60]. Further, bulling affects QOL negatively [52, 61]. The participants in our study reported suffering severe pain, which in itself affects QOL negatively [61]. Frequent pain hampers possibilities for managing school performance and leisure activities [62]. It is possible that in in addition to family problems, continuous pain and negative feedback from peers over time give the adolescents a negative feeling about themselves and reduce their self-image. We observed gaps between the adolescents’ idealized images of being successful and how they rated themselves. Closing or reducing these gaps might increase QOL [63].

### Strengths and weaknesses

The main author, who conducted the interviews, has specialized in psychiatric nursing. During the interviews, she may have used psychiatric interview techniques which revealed more in-depth information, in comparison to other interview approaches. Also, the fact that the interviewer was female may have made the girls, who made up the majority of the interviewees, feel more comfortable and willing to disclose information than the boys. A potential limitation of the present study is that, of the 19 participants, only 3 were boys. Perhaps additional, different viewpoints would have been revealed if more boys had been included in our sample. Another limitation was the ethical requirement to inform parents of the study. This may have excluded students who did not want to fully disclose their OTCA use to their parents. Further, we did not recruit adolescents from all ten schools and there may be other adolescents with special experiences to which we did not have access. We do not believe our results are geographically biased. A previous study failed to find a difference in the consumption of OTCAs among adolescents living in urban regions compared to those in rural regions of Norway [2]. We were also aware that the background, position, and preconceptions of the researchers could potentially affect the focus and perspectives, and the research group voiced this as an important consideration. Despite potential limitations, we believe this study provides useful information on adolescents who have frequent pain and a high use of OTCAs. If the findings are recognized as useful, they may be transferrable within the framework of qualitative research [64].

## Conclusions

Childhood experiences affect adolescents’ pain experience and OTCA use. Appraisal of pain and use of OTCAs may be learned. Expressing pain may also provide a way for adolescents to receive support from caregivers. Furthermore, expressing pain can serve as a proxy and an ‘acceptable’ way of revealing unpleasant thoughts and feelings. Indeed, experiences early in life might influence the regulation of stress systems in ways that shape one’s future responses with regard to pain expression. Coping with stress by avoiding it or hiding its symptoms can perpetuate or even amplify underlying problems. These complex mechanisms may reduce adolescents QOL and must be considered in order to identify, map and support adolescents with severe pain and a high consumption of OTCAs.

Our findings apply to the practice of both school nurses and general practitioners supporting adolescents. Health professionals should be aware that frequent pain and high use of OTCAs may be caused by complex and serious conditions that the adolescents may attempt to conceal. Social support is found to increase optimism and improve health in early adolescence and this may be a target for an intervention [65]. The adolescents may need a stronger network of assistance to deal with pain and the use of OTCAs. Future research and interventions targeting frequent pain and high OTCA use among adolescents might want to consider adolescent knowledge about analgesia in addition to networks of social support for individuals experiencing pain.
